# Needs, barriers and facilitators of older adults towards eHealth in general practice: a qualitative study

**DOI:** 10.1017/S1463423620000547

**Published:** 2020-12-02

**Authors:** Johannes William Vergouw, Hanneke Smits-Pelser, Marijke C. Kars, Thijs van Houwelingen, Harmieke van Os-Medendorp, Helianthe Kort, Nienke Bleijenberg

**Affiliations:** ^1^Student Nursing Science, Faculty of Medicine, University Medical Center Utrecht; ^2^Julius Leidsche Rijn Primary Health Centres Utrecht; ^3^Department Julius Center for Health Sciences and Primary Care, University Medical Center Utrecht; ^4^Research group Technology for Healthcare Innovations, University of Applied Sciences Utrecht; ^5^Saxion, University of Applied Sciences, School of Health, Deventer/Enschede; ^6^Research Group for the Chronically Ill and Elderly, Faculty of Health Care, University of Applied Sciences Utrecht; ^7^Department of Nursing Science, Julius Center for Health Sciences and Primary Care, University Medical Center Utrecht

**Keywords:** community-dwelling older adults, eHealth, online applications, primary care, qualitative study

## Abstract

**Background:**

The strain on health care services is increasing due to an ageing population and the increasing prevalence of chronic health conditions. eHealth could contribute to optimise effective and efficient care to older adults with one or more chronic health conditions in the general practice.

**Aim:**

The aim of this study was to identify the needs, barriers and facilitators amongst community-dwelling older adults (60+) suffering from one or more chronic health conditions, in using online eHealth applications to support general practice services.

**Methods:**

A qualitative study, using semi-structured followed by think-aloud interviews, was conducted in the Netherlands. The semi-structured interviews, supported by an interview guide were conducted and analysed thematically. The think-aloud method was used to collect data about the cognitive process while the participant was completing a task within online eHealth applications. Verbal analysis according to the Chi approach was conducted to analyse the think-aloud interviews.

**Findings:**

A total of *n* = 19 older adults with a mean age of 73 years participated. The ability to have immediate contact with the GP on important health issues was identified as an important need. Identified barriers were non-familiarity with the online eHealth applications and a mismatch of user health needs. The low computer experience resulted in non-familiarity with the online eHealth applications. Faltering applications resulted in participants refusing to participate in the use of online eHealth applications. Convenience, efficiency and the instant availability of eHealth via applications were identified as important facilitators.

**Conclusion:**

To improve the use and acceptability of eHealth applications amongst older adults in the general practice, the applications should be tailored to meet individual needs. More attention should be given to improving the user-friendliness of these applications and to the promotion of the benefits such as facilitating older adults independent living for longer.

## Introduction

In 2019, the number of people aged 60 years and older was 1 billion (World Health Organization, 2019a). This number will increase to 1.4 billion by 2030 and 2.1 billion by 2050 (World Health Organization, 2018). The need for health care services is increasing substantially due to the ageing population and the increasing prevalence of multimorbidity (Parker *et al.*, 2014). Multimorbidity can be defined as the co-occurrence of multiple chronic or acute diseases and medical conditions, which require complex and continuous care (Mercer *et al.*, 2016).

The number of older adults with multimorbidity who are living alone is growing (Van den Berg *et al.*, 2012). Most older adults aim to live independently at home for as long as possible (Van Duin *et al.*, 2013; Doekhie *et al.*, 2014; Bähler *et al.*, 2015). The primary health care system is continuously being challenged to provide optimal and efficient care to older adults with multimorbidity, due to the substantial increase within this age group (Doekhie *et al.*, 2014; Bähler *et al.*, 2015). Older adults with multimorbidity visit a general practitioner (GP) approximately 15.7 times annually compared to 4.4 times a year for adults without chronic conditions (Bähler *et al.*, 2015). Furthermore, a higher age is associated with a higher number of GP consultations (Bähler *et al.*, 2015).

Health care innovations such as eHealth can contribute to more cost-effective care for the older adults (Livingstone & Solomon, 2015). eHealth, is defined as, the use of Information and Communication Technologies (ICT) for health (World Health Organization, 2019b). eHealth can facilitate health services to support and increase the level of health care access amongst health care providers (Fairbrother *et al.*, 2014). The Dutch Government aims to provide 75% of older adults with eHealth access in the near future and thereby reducing the growing health care costs (MVWS, 2014; Rijksoverheid, 2015). In this study, eHealth refers to online applications for use in general practices that can support and increase services available and access to the GP irrespective of time or location. Currently, 63.1% of older adults are using or intend to use online applications and 15.9% are not willing or able to use eHealth applications (Robben *et al.*, 2012; Foster & Sethares, 2014; Peek *et al.*, 2014; Currie *et al.*, 2015; De Veer *et al.*, 2015). Several other studies also identified different barriers and facilitators amongst older adults in using these applications.

Reported personal barriers that restrain the older adult from using eHealth are the lack of technological literacy (Foster & Sethares, 2014; Peek *et al.*, 2014; Currie *et al.*, 2015), impairments to their sight or hearing (Foster & Sethares, 2014; Currie *et al.*, 2015) or misinterpretation of information exchanged via online services (Jung & Loria, 2010). Other barriers associated with technological applications are the high costs involved and the preconceived idea that people wearing, for example, an alarm button, maybe considered frail (Peek *et al.*, 2014). Important facilitators of eHealth that have been reported are time flexibility of health care provision (Robben *et al.*, 2012; Currie *et al.*, 2015), improved health care access for patients with functional disabilities (Currie *et al.*, 2015) and a reduction in consultation time (Jung & Loria, 2010).

To facilitate and increase the use of eHealth applications amongst older adults visiting the general practice, it is necessary to explore their perspectives on eHealth and identify more (cost) effective healthcare (Thompson *et al.*, 2011; Van den Berg *et al.*, 2012; Heart & Kalderon, 2013; Steele Gray *et al.*, 2014; Greenhalgh *et al.*, 2015; Vermeersch *et al.*, 2015). However, little is known about what may hinder, facilitate or promote the use of eHealth applications amongst older adults in the GP setting.

Therefore, the aim of this study was to identify the needs, barriers and facilitators amongst community-dwelling older adults (60+) suffering from one or more chronic health conditions, in using online eHealth applications to support general practice services. These online applications may support and increase the level of health services available to the older adults, such as being able to contact their GP practice at any time and from anywhere.

## Design and methods

### Design

A qualitative study was conducted to explore in-depth and understand the experiences and perspectives of older adults with multiple chronic conditions when using online eHealth applications in the general practice, with special focus on needs, barriers and facilitators (Creswell, 2007). Semi-structured qualitative interviews were conducted to build up a rapport and to gain information from the participants’ perspectives about eHealth applications (Holloway & Wheeler, 2010). A think-aloud method was conducted afterwards to explore how participants conduct a task within the eHealth applications, while they voiced their minds during this process (Polit & Beck, 2012). This method was used to better understand how the participants responded while using the specific applications to determine what barriers and facilitators they perceived when using it.

### Setting, recruitment and participants

Participants were recruited from Julius Leidsche Rijn Primary Health Centres (JHC), an academic primary care organisation with 5 general practices and 30 GPs in the city of Utrecht, the Netherlands. JHC provides integrated primary healthcare to approximately 42.000 inhabitants, of whom 8.7 % are above 60 years of age (Gemeente Utrecht, 2017). At this general practice, several online eHealth applications are being used by the health care professionals such as GP and practice nurses and patients.

First, eligible participants aged 60 years and over with one or more chronic conditions were invited by the GP to consider participating in the study. Subsequently, they were approached by telephone. If a participant was willing to participate, an information letter with an informed consent form was send by post or email. Within five working days, the participant was contacted by telephone again to explain the study procedures and to schedule an appointment for the interview at the participant’s home.

Inclusion criteria were able to speak Dutch, living independently at home, Internet literate and non-users of the online eHealth applications in general practice. Internet-literate was defined as someone with experience of using the internet and is at least capable of using email. To work safely with the online eHealth applications, persons need to have a Digital Identity (DigiD) number. A DigiD is a safe system provided by the Dutch Government for citizens to log into websites of government agencies and health care organisations. Participants were excluded if they were in a poor physical condition or suffering from cognitive disorders.

### eHealth applications

The experiences of participants with the following eHealth applications were assessed: (1) e-Consultation (secure messaging): patients can consult a health care professional safely online via the Internet, (2) e-Appointment: patients can schedule an appointment via the Internet, (3) e-Prescription: patients can order medications via the Internet, (4) e-Lab: patients can view their own lab results and (5) e-File: patients have access to their medical file within the general practice. These applications were accessible via a private Internet portal on the Internet website of the general practice. Patients can log in with their DigiD and can view their own information or have the opportunity to send a digital secure message. The applications are available everywhere as long as there is Internet access (Pazio, 2015).

### Data collection

Prior to the data collection, we predetermined the concepts needs, barriers and facilitators based on the literature and practice relevance. Needs were defined as the need or desire according to one’s skills, knowledge and resources in order to utilise the online eHealth applications in the general practice. Barriers were defined as the aspects that prevent one from using online eHealth applications. Facilitators were defined as aspects that facilitate and support older adults in actually using the online eHealth applications.

The semi-structured interviews were conducted to build up a rapport and is followed by the think-aloud interviews. The interviews were recorded and transcribed verbatim to ensure the validity of the data analysis (Halcomb & Davidson, 2006; Boeije, 2010). A think-aloud method is a method where the researcher asks the participant to respond to the questions while the participant is using the technology, in this study the eHealth application. This method collects data about the cognitive process while the participant is completing a task while using the online eHealth applications. During this process, in-depth data were collected on how adults perceive and use the online eHealth applications (Polit & Beck, 2012). This provides insight into what a participant already knows and or their lack of knowledge, while participants are using the applications (Creswell, 2007). The think-aloud method has been used in a comparable area before (Luger *et al.*, 2014). To improve the validity of the data, data triangulation was applied by using the two different interview methods to better understand and comprehensively examine the needs, barriers and facilitators (Holloway & Wheeler, 2010).

### Demographics

Prior to the interview, baseline characteristics such as age, sex, living situation (alone or with others), educational level (low: primary school or lower, average: higher educated than primary school and high: higher vocational education or university), number of chronic conditions and number of general practice visits per year were collected using a short questionnaire.

### Semi-structured interviews

The interview guide consisted of three main topics: needs, barriers and facilitators (Table 1) and was based on previous studies that explored these topics (Jung & Loria, 2010; Robben *et al.*, 2012; Heart & Kalderon, 2013; Foster & Sethares, 2014; Peek *et al.*, 2014; Steele Gray *et al.*, 2014; Currie *et al.*, 2015). During the iterative process of data collection and analyses, minor refinements to the interview guide were made.

**Table 1.** Interview guide semi-structured interview

Subject	Question	Question for participant
Opening question	What can you tell me about the current eHealth (digital) applications (or other computer programs), which are used in general practice?	What can you tell me about the current digital computer applications, which are used in general practice (and are available for patients)?
Needs	Which needs or desires do older adults have within general practice in accordance with skills, knowledge and resources in using the online eHealth applications?	• What do you think of the e-Consult application, the e-Appointment application, the e-Prescription application, the e-Lab application and the e-Dossier application? Why?
	**eHealth:** The use of new information and communication technologies, with emphasis on Internet technology, to support or improve health and healthcare (13).	• What would you like to do via the Internet to contact the GP?
		• Do you lack applications or useful features, related to the GP, which you would like to use digitally?
	**e-Consult:** (patients can consult a health care professional safely via the Internet)	• Which requirements must the applications meet, which would entice you to use the programmes? Why?
	**e-Appointment:** (patients can book an appointment via the Internet)	• Do you think that you have sufficient knowledge to use the application? Do you have people around you that could support you in using the application? Do you ask others for help? Do you receive help swiftly?
	**e-Prescription:** (patients can order medication via the Internet)	
	**e-Lab:** (patients can view their own lab results)	
	**e-Dossier:** (patients have access to their general practice medical file)	• Do you use other health-related digital applications (apps on the phone)? Why?
		• Do you have a preference for a specific device when using the applications, like a computer or a tablet? Why?
Facilitators	What are the characteristics of older adults that facilitate the use of eHealth?	• Do you personally understand the advantages of using applications? Which or which not?
		• In a non-emergency situation, would it be easier for you to contact the GP via the applications, without making a phone call, or by direct contact?
Barriers	Which aspects do older adults experience as barriers to using the eHealth applications? Or what restrains an older adult in using eHealth applications?	• In daily life you are known to use digital applications such as email, Internet banking, using digital identity (DigiD) or arranging health insurances online. Can you explain what restrains you from using the online eHealth applications in general practice?
		• What could help you in using the online eHealth applications?
		• Would you like to use the online eHealth applications in the future? Why or why not?
		• How do you experience the provided information about the applications from the GP?
		• What should the GP do to enhance the use of the applications by patients? What exactly?
		• Do you see any disadvantages regarding the use of digital applications within a general practice setting?
Termination	Are there still matters, which the respondent would like to discuss?	• Do you think that these applications contribute to living independently at home?
		• Do you think that eHealth in general contributes to enhanced health care provision and independent living at home? Why?
		• Do you want to discuss something else regarding eHealth in a general practice setting?

### Think-aloud

After the semi-structured interview, the think-aloud interview was conducted while participants executed tasks associated with two online eHealth applications. Participants were asked to choose one of the online applications. The second eHealth application was chosen by the researcher to ensure that all the applications were addressed throughout the study. During the think-aloud interview, the participants were invited to speak their thoughts aloud, while performing their eHealth task.

### Ethical approval

This study was conducted according to the principles of the Declaration of Helsinki (World Medical Association, 2015). All participants provided written informed consent prior to the interviews. In accordance with the University Medical Centre Utrecht Research ethics committee guidelines, the study did not apply to the Medical Research Involving Human Subjects Act as participants were not subjected to treatment or required to follow any certain behavioural strategy.

### Data analysis

During the thematic analysis the three main concepts needs, barriers and facilitators were used as starting points for coding the data.

The interviews were transcribed verbatim and read to assist in the identification of relevant fragments and confirmation of identified themes (Holloway & Wheeler, 2010). Subsequently two specific methods, a thematic analysis (Braun & Clarke, 2006) and verbal analysis (Chi, 1997) were used to unravel the data. Thematic analysis according to Braun & Clarke (2006) was used to analyse the semi-structured interviews. Thematic analysis is a flexible method for identifying themes within the data of a qualitative study. Thematic analysis aims to organise the verbal data and support it with interpretation. The analysis was performed following three steps. Meaningful segments were coded. These codes were assigned to themes and these themes were reviewed. Consequently, themes were defined, clustered and if necessary refined by the researchers (Braun & Clarke, 2006). After 10 interviews, no new themes were identified. In order to increase the interrater reliability, three interviews were analysed with two researchers together. All themes were discussed to establish consensus. Conflicting interpretations were discussed until consensus was reached.

To analyse the think-aloud interviews, verbal analysis according to Chi (1997) was used. Verbal analysis is an appropriate method since interactions such as movements can be incorporated into the transcripts. This analysis was performed using the following steps. First, data were segmented into meaningful units in relation to the needs, barriers and facilitators. Second, a coding scheme of the meaningful units was developed to analyse the think-aloud interviews (Chi, 1997). Within this scheme, an existing classification of problem types was used (Van den Haak *et al.*, 2007) (Table 4). Third, topics were identified and coded from the different segments. Finally, patterns between the coded segments were identified to compare each segment in view of arrangement (Chi, 1997). NVivo version 11 was used to analyse the data.

## Results

**Figure 1.**
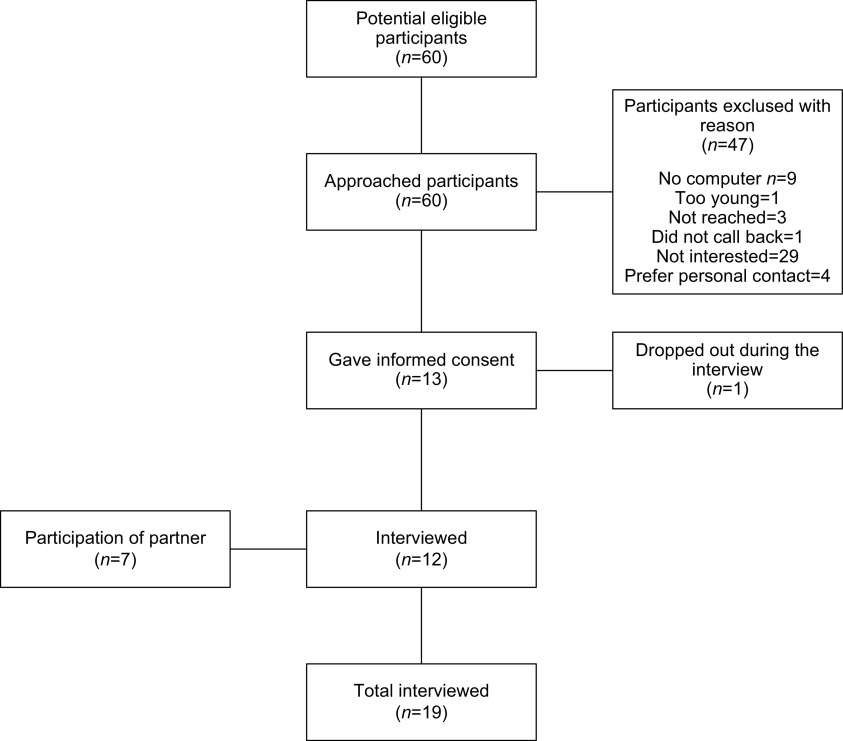
Flowchart recruitment.

In total, 60 eligible participants were approached by telephone to participate, and 47 of these refused to participate due to different reasons such as; preferring face-to-face contact, uninterested in using digital applications or not being Internet literate (Figure 1). In total, 13 participants were subsequently interviewed. One participant withdrew from the study during the interview due to lack of interest. Twelve participants were included in the study. Seven of the 12 participants were married. Their partners also agreed to participate, resulting in a total number of *n* = 19 participants. The mean age was 73 (SD 5.3), and 9 (47%) were female. All participants were moderate or highly educated, 13 (68%) were married and the median number of chronic conditions was 1 (IQR: 0–2) (Table 2).

**Table 2.** Characteristics of the participants

	*n* = 19
Age, mean (SD)	73.26 (5.3)
Female, *n* (%)	9 (47.4)
Marital status, *n* (%)	
Married	13 (68.4)
Unmarried together	2 (10.5)
Divorced	1 (5.3)
Widowed	3 (15.8)
Having children, *n* (%)	19 (100)
Educational level, *n* (%)^a^	
Low	1 (5.3)
Moderate	10 (52.6)
High	8 (42.1)
Number of chronic diseases, median (IQR)	1 (0–2)
Number of GP visits a year, mean (SD)^b^	3.26 (2.68)

a Low: primary school or less. Moderate: more than primary school. High: higher vocational education or university.

b Based on the question: ‘How many times a year do you visit the GP?’

**Figure 2.**
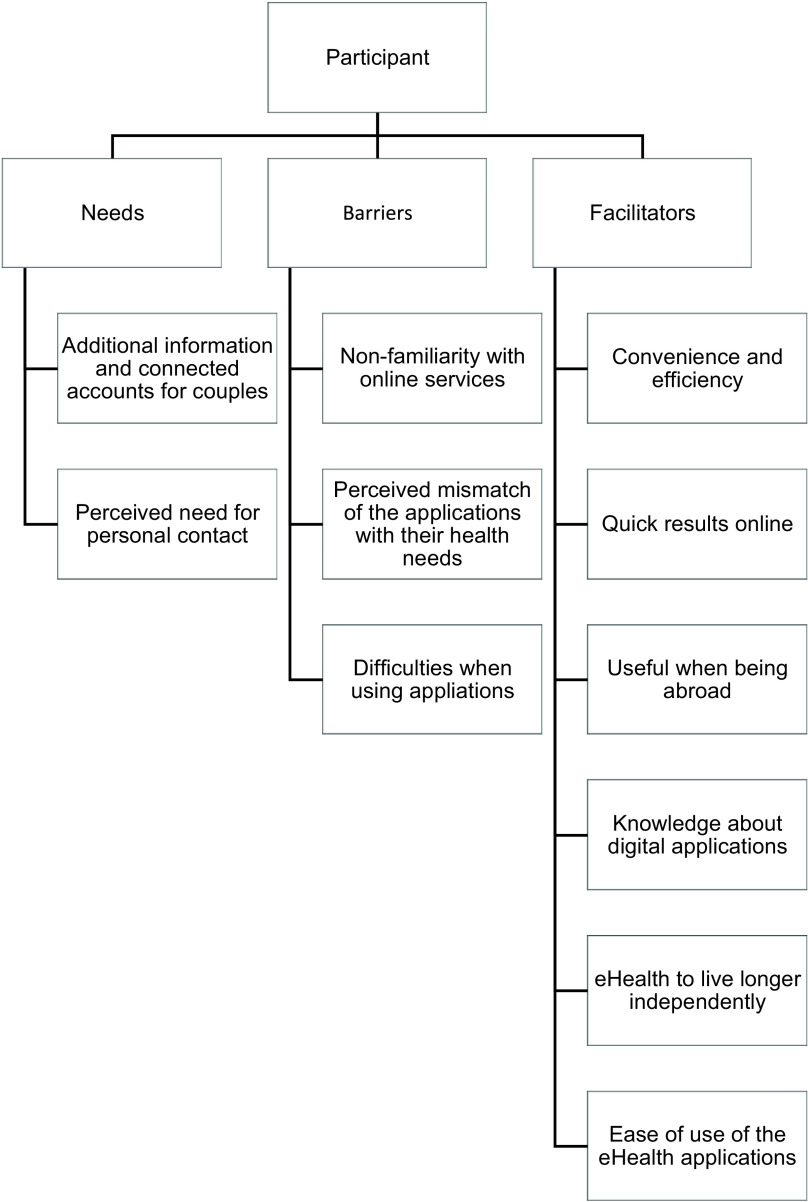
Themes per category.

The semi-structured interviews lasted approximately 60 minutes and the think-aloud interviews approximately 20 minutes. After 10 interviews, saturation was reached and no new themes were identified from the data. For extra validity, two extra interviews were conducted. Four–five themes per category were identified (Figure 2 and Table 3). The results of the interviews were categorised under the three main topics, needs, barriers and facilitators.

**Table 3.** Main themes and subthemes of facilitators, barriers and needs

			Source	
Category	Main themes	Subthemes	Interview	Think-Aloud
**Needs**	***Additional information and connected accounts for couples***	Additional desires for (personal) use	X	
		Specific content needed with the applications	X	
		Lack of information *(Completeness)* ^a^		X
		Way of promoting applications	X	
	***Perceived need for personal contact***	Triage by medical receptionists	X	
		Preference for personal contact	X	X
**Barriers**	***Non-familiarity with online services***	Threshold to start with applications	X	
		Negative experiences in the past with digital applications, which avoid usage	X	
		Log in problems and problems with DigiD^b^, which causes resistance in using applications	X	
		Lack of knowledge about the applications	X	
		Not having the opportunity to easily ask for help	X	
	***Perceived mismatch of the applications with their health needs***	Little contact with GP	X	
		Added value of applications is not recognised	X	
		Does not think that applications could support to live independently	X	X
		Personal factors, which avoid using applications	X	X
		Motivation for not using applications	X	X
	***Difficulties when using applications***	Participant does not understand something *(Comprehension, Completeness, Data entry)* ^a^		X
		Structure is unclear *(Structure)* ^a^		X
		Layout is unclear *(Graphic design, Relevance)* ^a^		X
		Visibility within the applications is unclear *(Visibility)* ^a^		X
		Problems during usage caused by the participant		X
**Facilitators**	***Convenience and efficiency***	(personal) motivation in using applications	X	
		Extra possibilities for contact	X	
		Use of applications can amplify contact with a health care provider	X	
		Ease of use for participants		X
		Pleasant informative for participants		
	***Quick results online***	Availability of results at any time anywhere	X	
		Recalling results	X	
	***Useful when being abroad***	Availability of applications abroad	X	
		Communication with own GP when being abroad	X	
	***Knowledge about digital applications***	Sufficient knowledge to work with the applications	X	
		Knowledge of the existence of the applications	X	
		If they get stuck with the application, they figure out themselves how the application works	X	
		Receiving assistance with the applications	X	
		Knowledge of how the computer works, eventual in combination with health	X	
		Participant will operate independently within the applications, despite something is not quite clear *(Structure)* ^a^		X
		Comprehension of the working of the applications without much assistance		X
		Knowledge of the older adult at the beginning of the use of the application		X
	***eHealth to live longer independently***	Believe that applications and digital resources can contribute to live longer independently	X	
		A look into the future	X	
		Usage in the future		X
	***User-friendliness and ease of use***	Easily logging on	X	
		Convenient arrangement and properly working application	X	
		Desires in relation to structure *(Structure)* ^a^		X
		Desires in relation to design *(Graphic design)* ^a^		X

a Refers to the classification of problem types by Van den Haak *et al.* (2007).

b DigiD: Digital Identity provided by the Dutch Government.

**Table 4.** Existing classification or problem scheme according to Van den Haak *et al.* (2007)

1. Comprehension	Participant finds that the information on the site is no clear or applicable: he or she experiences syntax problems; he or she finds the choice of vocabulary problematic.
2. Relevance	Participant feels that certain information should not be included or should be reduced.
3. Completeness	Participant feels that information is missing or more elaboration is needed.
4. Structure	Participant finds that the order of information is problematic or that the structure is not clearly signalled.
5. Formulation	Participant does not appreciate a particular formulation.
6. Graphic	Participant does not appreciate layout or illustrations.
7. Correctness	Participant detects a violation of syntax, spelling or punctuation rules.
8. Data entry	Participant does not know how to enter data on the site.
9. Visibility	Participant fails to spot a particular link, button or piece of information on the site.

### Needs

Several needs were identified from both the interviews regarding the eHealth applications. Older adults emphasised the need and desire to possess adequate skills, knowledge and resources in order to utilise the online eHealth applications with their home devices. Participants mostly preferred using the computer or laptop instead of a tablet due to the screen size. Although, others preferred to use tablets, due to its speed and quick boot issues:

‘*Well, firstly I always have the tablet next to me. It is faster, than booting up the computer, is terrible and takes a while.’ (R14, female, 75 years)*

#### Additional information and connected accounts for couples

Participants expressed the need for a paper manual containing extra information on the use of the applications. One participant suggested the possibility of constructing joint accounts for couples, so that one partner may operate the applications for them both.

Moreover, participants expressed their needs to be better informed about the availability of the applications either by letter, information sheets, advertisements in the local newspaper or email. One participant suggested that repeated advertising could be useful to increase the level of awareness amongst older adults.

#### Perceived need for personal contact

Eight participants preferred personal contact, although seven of them already had expressed the advantages associated with using the eHealth applications. However, certain situations such as the urgency of a complaint, cannot be assessed online by the GP. Besides, personal contact was preferred because it offered reassurance and the ability to ask questions right away. Furthermore, other personal factors included the fear of making mistakes or the uncertainty about a message being delivered to the right recipient:

*‘And yes, if I do it myself [ordering medications using the eHealth application], then I am uncertain if I did it correctly.’ (R13, male, 69 years)*

Additionally, some participants expected that their use of the applications would decrease in the future, as the need for personal contact would increase as participants are getting older. One participant explained this as follows: *‘If I have limitations in daily functioning and I am alone, I think I will have no need for applications. Then I prefer to make a phone call, to have more personal contact.’ (R15, male, 69 years)*

### Barriers

Several barriers were identified by the participants that limed the usability of the eHealth applications.

#### Non-familiarity with online services

The main reason for not using the eHealth applications was due to unfamiliarity with the applications. Participants did not grow up with digital devices, and therefore needed more time to learn how to use the applications:

*‘But we did not grow up with the computer. I would rather make a phone call to arrange an appointment and prefer to talk face-to-face to the physician.’ (R8, female, 71 years)*

Three participants had difficulty in determining whether their medical complaint was urgent enough to warrant a visit to the GP and was therefore not using the e-Consult. They felt reassured have spoken to the doctor’s assistant or receptionist by phone. One participant declared not being able to define the urgency of the situation:

‘*I think that is very difficult. Where do you draw the line between using e-Consult or calling the GP practice directly when you are sick?’ (R10, female, 80 years)*

#### Perceived mismatch of the applications with their health needs

Some were neither open, nor did they envision any benefits of using the application. They stated that they always had enough time and opportunity to contact the GP when it was necessary. Four participants found the use of the eHealth applications unnecessary as they had little or no contact with the GP, and therefore saw no added value in having or using them.

Additionally, personal factors also played a role in avoiding the use of applications such as disliking the current layout, not wanting to wait for an answer and concern about what might happen to their private medical history information while using these eHealth applications. Five participants mentioned that they had no need for further insight into their health status, as it is already known to them. Besides, other organisations such as pharmacies or thrombosis services already provided participants with their required medical information.

Furthermore, three participants explained their preference to personally visit their general practice instead of using the applications due to the closeness of their home to the practice itself. For this reason, one participant found the applications nonsense:

‘*I already indicated that this is nonsense, because it is a short walk to the general practice for me.’ (R10, female, 80 years)*

#### Difficulties when using applications

Although the participants were able to use and interact with the applications, during the think-aloud interviews, it was noted that comprehension, completeness, data entry and structure were issues that they experienced difficulties with. When it was unclear to the participants what to do next during the use of the applications, they perceived a limited understanding of the applications (comprehension) and how to enter the right information (data entry) as major impediments. Some stated that the structure of the page was unclear, not always knowing which buttons should be used to continue within the application. Visibility within the applications and proper placement of action buttons was sometimes indicated as unclear.

During the think-aloud interview, it was observed that participants did not take sufficient time to read the webpage or the notifications which appeared on the screen. Participants missed information on how to continue in the application, not realising that by scrolling down the page this would bring them to the following button. A participant remarks:

*‘I will just have a try, of course. Arranging an appointment, that is clear but adding an action [the option to open one of the other applications within the screen], I don’t know what that is exactly. This is unclear, it is too difficult for me.’ (R4, male, 75)*

Furthermore, other barriers contribute to the reluctance to use the applications such as past negative experiences with digital applications, difficulty with the log in and too many screens making things complex. Participants stated they could not easily ask someone for help and nine participants experienced some lack of practical information about the applications and their functionality.

*‘I don’t know how long it will take to receive an answer, or who will answer. I want to have assurance straight away. When you send the e-Consult, then you never know what will happen with it.’ (R4, male, 75 years)*

### Facilitators

Six facilitators emerged from the interviews and think-aloud interviews that facilitate the use of the applications.

#### Convenience and efficiency

Most of the information provided in the applications was found to be sufficient. Specifically, the notification in the pop-up screen to contact the general practice by phone in case of emergency along with the replies from the GP via the applications were appreciated.

Most participants mentioned reasons favouring the use of the applications, the most common reasons were being the convenience and efficiency of the applications. Participants stated that they could use the applications anywhere, any time and at their own convenience, independent of the availability of staff at the general practice who answer the phone. One participant noticed:

‘*Yes, if call in the morning, you can be waiting on hold for quite a while before you get an answer.’ (R15, male, 69 years)*

#### Quick results online

Participants stated that access into their e-File and e-Lab could contribute to quicker access to results and information along with the ability to compare the information. The possibility for participants to use the applications at their own leisure was expressed as an advantage. One participant explained:

‘*Yes, the advantage is that I can go on my device at my own leisure in my own time, without being limited to the allotted 10 minutes of the physician time. At my leisure I can review previous results and information.’ (R11, male, 69 years)*

#### Useful when being abroad

One participant mentioned that these applications could be very useful abroad, for example, during a holiday. Despite the difference in time zones it is possible to communicate with their own GP, avoiding high phone costs.

#### Knowledge about digital applications

Most participants stated they had sufficient knowledge on how to use the applications, which was acquired from other computer experiences, through trial and error and by using other digital applications. One participant explained:

‘*I will figure it out myself, no matter how long it might take. I will click all the buttons until I understand what they meant for.’ (R3, male, 82 years)*

The think-aloud interview showed that all the participants understood how to use the applications and completed most tasks themselves within 5 minutes requiring little or no help from the researcher.

#### eHealth to live longer independently

Two participants believed that the current applications supported in the general practice could contribute to living a longer independent life. They stated that contact with the GP would be improved and that direct contact was not always necessary. Therefore, online eHealth applications are an easy way to get and stay in contact with the GP. Furthermore, two participants believed that this extra opportunity to get into contact with the GP could contribute to improving the relationship with the GP more so than in the past. One participant expressed this as follows:

‘*I believe that contact with the general practitioner will be strengthened by using the applications more frequently even though you can’t see her. Thus, easy to get in contact with the GP and get a proper assessment on my current health status.’ (R5, female, 68 years)*

## User-friendliness and ease of use

To facilitate the use of the applications, quick and easy access was the most important requirement according to the participants. One participant responded:

‘*Accessibility is important to me. You should be easily able to log on using your username and password.’ (R15, male, 69 years)*

All participants stated that the applications have to be designed clearly and simply. In addition, applications need to function properly to avoid participants from abandoning the use of them. One participant stated that he gets furious if an application does not function properly:

‘*But I really get furious when things do not work properly. That is difficult for me. I try but when it does not work I tend to give up.’ (R6, male, 72 years)*

Further two participants desired a larger letter font.

Six participants praised working with the applications because of their clarity and ease of use. It was regarded that the main screen was clear and the system was easy to operate:

‘*Yes, how to operate the applications was all clearly indicated, there was nothing wrong there.’ (R8, female, 71 years)*

## Discussion

This study provided unique insights into needs, barriers and facilitators of community-dwelling older adults using eHealth applications in the general practice. From the semi-structured and think-aloud interviews, it was observed that personal contact is an important need for feeling reassured. That non-familiarity with online services and mismatch with health care needs turned out as barriers. And the ability with quick access to results along with more possibilities for easy contact with the GP facilitates in using the eHealth applications.

Several factors regarding the use of digital applications in this study correspond with existing literature such as that eHealth applications provide flexibility for participants in daily living (Robben *et al.*, 2012; Currie *et al.*, 2015), reduction in waiting time (Jung & Loria, 2010), easy access to healthcare without having to travel anywhere (Currie *et al.*, 2015), and user-friendliness (van den Berg, Schumann *et al.*, 2012; Foster & Sethares, 2014). In addition, the current study provided more insight into the perspectives of older adults in terms of knowledge in eHealth applications and device preferences. The identified barriers, which were in line with previous studies, were misinterpretation of information (Jung & Loria, 2010) and preferring face-to-face contact (Robben *et al.*, 2012). However, only one study focused on frail older adults (Robben *et al.*, 2012). The results of this study provide insight into the perceived usefulness, barriers and the associated patient needs when using digital applications. Due to the pre-conditioned population, practical factors, such as not being Internet literate or physical impairment were not addressed (Foster & Sethares, 2014; Peek *et al.*, 2014; Currie *et al.*, 2015).

Some of the participants acknowledged that the eHealth applications are easy to use when they are abroad and that due to the application, they are not dependent on the availability of health care staff. This is in line with the findings from another study that found that older adults living at home experienced more control over their own healthcare after using an online health management tool (Zettel-Watson & Tsukerman, 2014). Participants were more likely to use applications if they experienced convenience and efficiency during use. Alternatively, it was noted that non-users did not see the need for using applications (Zettel-Watson & Tsukerman, 2014), which is in line with our current study. Also, the Technology Acceptance Model (TAM) described that perceived usefulness and perceived ease of use are important factors for using digital applications (Davis, 1989). The TAM displays the essential relationship between perceived usefulness and perceived ease of use for a successful adoption of digital applications in users (Davis, 1989). This indicates that it is important to focus on the perceived usefulness and perceived ease of use within the promotion of eHealth applications. This model possibly explains why participants’ answers were sometimes somewhat ambiguous in using the applications.

The results of our study indicate that ‘one size fits all’ approach does not apply to older adults when using online eHealth applications at home. A recent study suggests that eHealth implementation strategies have to be improved and must meet end-user needs. Simply developing and offering applications is insufficient and does not fit current practice (Ossebaard & Van Gemert-Pijnen, 2016). Co-creation, as a user-centred design, should be considered within the development of the implementation and dissemination of eHealth applications as well (Korpershoek *et al.*, 2020).

### Strengths and limitations

A strength of this study was the fact that two methodological approaches were used simultaneously, semi-structured interviews and the think-aloud method. The advantage of using both methods was to gain a comprehensive insight into the participants’ experiences and perspectives regarding the use of digital applications. The think-aloud interviews results yielded concrete thoughts and suggestions about the specific applications while using the eHealth applications (Polit & Beck, 2012). Data triangulation of these two methods (Chi, 1997; Polit & Beck, 2012) was used which strengthened the internal validity and resulted in the main themes.

Some limitations need to be considered. First, selection bias cannot be ruled out as only the most interested participants gave their consent. Although the studied population was comparable with the current Dutch population based on age, marital status, educational level and health status (Boer, 2006; Van Solinge, 2015). Participants were recruited from only one general practice setting, making generalisation difficult. Second, it is important to note that the Internet accessibility in the Netherlands for older adults is rather accessible. In 2017, Internet accessibility was 88.3% for older adults with an age of 65 years or over (Centraal bureau voor de statistiek, 2018), and therefore caution is required when generalising the findings of this study to countries with lower Internet accessibility. Third, in this study, most participants were willing to use the applications because the researcher had provided them with the necessary information on how to use them. Without this additional information, it is possible that these participants would still be non-users. Finally, we have interviewed couples together, so it is possible that they influenced each other.

The results of this study suggest that participants who are open to and will use the applications if these applications meet participants’ needs and expectations. With this knowledge, new applications should be tailored and designed based on the desired users’ needs, so that they can easily be embedded in current practice. To achieve this, it is therefore important to involve older adults in the development of new applications (Robben *et al.*, 2012; Kayser *et al.*, 2015; Robert *et al.*, 2015; Peek *et al.*, 2016). We observed that participants were possibly influenced by their GPs in using the applications, and social influence has been described as an important element in user acceptance of information technology (Venkatesh *et al.*, 2003). It is essential that health care practitioners have sufficient skills and knowledge to work with the applications and to provide their patients with the necessary information about on how to use the applications (Van Houwelingen *et al.*, 2015). Therefore, eHealth usage, including the facilitators, barriers and needs of end users, should become more embedded in the current health education programmes of future health care providers (Van Houwelingen *et al.*, 2016). To implement eHealth applications in clinical practice, further research is needed to determine optimal strategies to develop, implement and promote the use of these applications in clinical practice. Also, health care professionals should be adequately equipped and trained to use and explain these applications adequately to their patients (Van Houwelingen *et al.*, 2018).

## Conclusion

This qualitative study addressed important needs, barriers and facilitators in the use of online eHealth applications amongst older adults with chronic health conditions. Personal contact is addressed as an important need for feeling reassured. Non-familiarity with online services and mismatch with health care needs appeared to be barriers that affect the use of online eHealth applications by older adults. The ability to have quick access to results along with easy contact with the GP encouraged and strengthened the ability to allow older adults to live longer independent lives. A user-friendly interface supports older adults in using online eHealth applications. Attention is needed to invite older adults in the development process of new applications to ensure its feasibility and to ensure that their needs are fulfilled.
